# Solitary Pediatric Osteochondroma of the Spine With Cord Compression

**DOI:** 10.7759/cureus.23342

**Published:** 2022-03-20

**Authors:** Tania Mamdouhi, Prashin Unadkat, Morris C Edelman, Alan A Johnson, Carolyn Fein Levy, Mark A Mittler

**Affiliations:** 1 Pediatric Spine Surgery, Donald and Barbara Zucker School of Medicine at Hofstra/Northwell, Hempstead, USA; 2 Pediatric Neurosurgery, Cohen Children’s Medical Center, New Hyde Park, USA; 3 Pediatric Neurosurgery, Donald and Barbara Zucker School of Medicine at Hofstra/Northwell, Hempstead, USA; 4 Pediatrics, Pathology and Laboratory Medicine, Cohen Children’s Medical Center, New Hyde Park, USA; 5 Pathology and Laboratory Medicine, Donald and Barbara Zucker School of Medicine at Hofstra/Northwell, Hempstead, USA; 6 Radiology, Division of Neuroradiology, Long Island Jewish Medical Center, New Hyde Park, USA; 7 Neuroradiology, Donald and Barbara Zucker School of Medicine at Hofstra/Northwell, Hempstead, USA; 8 Pediatrics, Division of Hematology/Oncology and Cellular Therapy, Cohen Children’s Medical Center, New Hyde Park, USA; 9 Pediatrics, Division of Hematology/Oncology and Cellular Therapy, Donald and Barbara Zucker School of Medicine at Hofstra/Northwell, Hempstead, USA

**Keywords:** pediatric spine osteochondroma, cord compression, pediatric osteochondroma, spine osteochondroma, osteochondroma

## Abstract

Osteochondromas typically arise in the appendicular skeleton, with axial lesions occurring less commonly. Osteochondroma of the spine resulting in cord compression and symptomatic myelopathy is relatively rare. Most cases are reported in adolescents and adults. Consequently, there is a scarcity of literature regarding its occurrence in the pediatric population. We report the case of a cervical osteochondroma of C4-6 with cord compression in a nine-year-old girl. Surgical excision with laminectomy and laminotomy successfully resolved all neurologic deficits. A literature review revealed 27 cases of pediatric osteochondromas with cord compression, suggesting that these lesions are not as rare in the pediatric population as previously thought.

## Introduction

Osteochondromas (exostoses) are characteristically benign, with only 1-5% progressing to chondrosarcoma [1]. They appear as bony protrusions with a cartilaginous cap, most typically along the metaphysis of long bones. Solitary lesions are the most common in individuals diagnosed with osteochondroma. Conversely, hereditary multiple exostosis is an inherited form of osteochondroma with multiple lesions appearing throughout the body, accounting for 15% of cases [1].

Approximately 1-9% of osteochondromas involve the spine [2-4]. Although lesions typically project outwards, rare exophytic expansion into the spinal canal with cord compression has been increasingly reported throughout the last decade [2,4]. Spinal lesions pose a risk for cord compression and permanent neurologic complications and should be considered in the diagnostic evaluation of suspected myelopathy.

There are a limited number of case reports describing osteochondroma of the spine with cord compression in the pediatric population. The majority of lesions are described in adults, with an average age of occurrence being 35 years old [2]. Here, we present a case of pediatric osteochondroma of the spine with spinal canal expansion and subsequent cord compression. Due to previous reports of such lesions being a rare description in the pediatric population, we conducted a comprehensive, up-to-date review of the literature to increase clinical detection and help guide clinical decision-making.

## Case presentation

A nine-year-old female presented due to concern about a gait abnormality. Her parents noted a gait disturbance for approximately two years that had progressively worsened. Her perinatal and developmental history were unremarkable. She was evaluated two years previously for a fall on her right shoulder, but no workup was indicated due to the absence of any orthopedic or neurologic abnormalities. At presentation, she reported no pain, focal weakness, or limitation in function. Her physical examination revealed mild external rotation of the right lower extremity. She had a full range of motion in her neck. Examination of the spine showed no evidence of swelling or deformity. There was noticeable atrophy of the muscles in the right upper and lower extremity. Deep tendon reflexes on the right showed 3+ hyperreflexia, positive Babinski and Hoffman sign, as well as two to three beat ankle clonus. Examination of the left side revealed 2+ deep tendon reflexes, with an absent Babinski sign and no ankle clonus.

**Figure 1:**
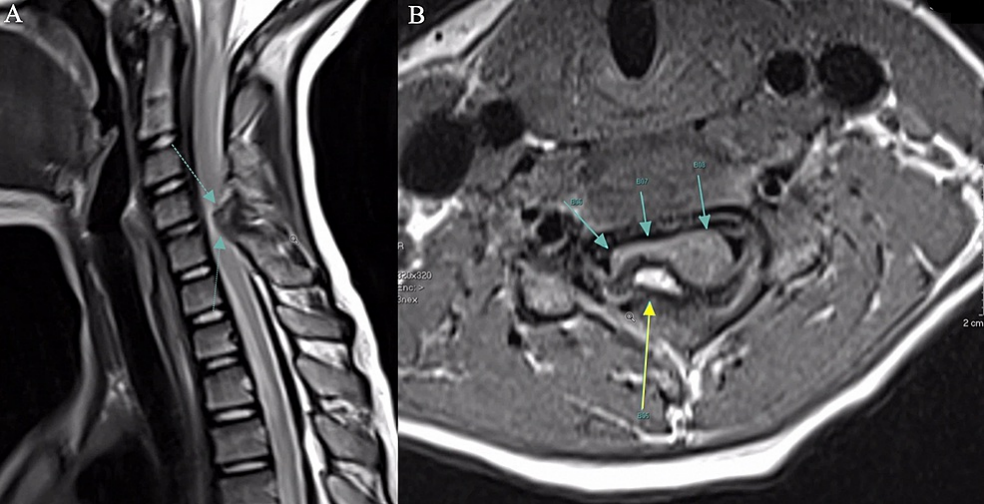
MRI cervical spine T2-weighted sagittal view (A) denoting bony expansile lesion with extension into the spinal canal (dashed arrow). MRI cervical spine T1-weighted axial view (B) showing bony lesion (solid blue arrows) and high-grade compression of the right aspect of the spinal cord (yellow arrow).

Magnetic resonance imaging (MRI) of the cervical spine showed an expansile bony lesion at the C4-5 levels with exophytic expansion into the spinal canal and compression of the right aspect of the spinal cord (Figures 1A, 1B). Follow-up computed tomography (CT) revealed a large pedunculated bony lesion arising from the lamina of C6 extending into C4-5 resulting in pronounced compression of the cord at the C4-5 level.

**Figure 2:**
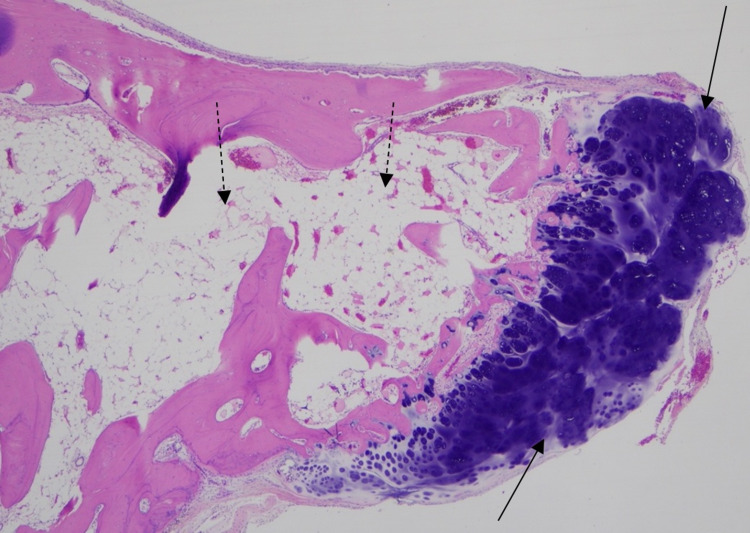
Histologic appearance of hematoxylin and eosin-stained specimen showing cartilage capped (solid arrow) osteochondroma with a central medullary space filled with adipose tissue (dashed arrow).

Right laminectomy of C5 and right laminotomy of C4 and C6 was conducted. Direct visualization revealed a calcified tumor that was removed. No evidence of destabilization was observed eliminating the need for fusion. Histopathology confirmed a cervical spine osteochondroma (Figure 2). Postoperatively, the patient was neurologically intact and ambulating. Postoperative MRI showed resolution of cord compression. A follow-up evaluation is planned to monitor for signs of recurrence.

## Discussion

The majority of meta-analyses and literature reviews to date have focused solely on osteochondromas of the spine, with some evaluating the presence of spinal cord compression and very few focusing on the presence of disease in the pediatric population. In general, most spinal osteochondromas are asymptomatic, with compressive myelopathy occurring infrequently. Typical symptoms and signs include hypesthesia, paralysis, gait abnormality, hyperreflexia, and pathologic reflexes [5]. Although males often comprise a higher proportion of osteochondroma cases reported in the literature, this rarely yields a significant difference in the reported case series [3,6]. To date, reports of pediatric osteochondromas with cord compression are limited to specific vertebral levels [7,8] or are described in brief case series [5,9], making it difficult to discern any pattern of disease for this population. Between 1969 and 2021, we identified a total of 27 cases of pediatric osteochondroma with spinal cord compression (Table 1).

**Table 1:** Literature review including reported cases of pediatric osteochondroma of the spine with cord compression.

Author	Year	Age (years)	Sex	Location of the lesion
Hickey [10]	1969	12	F	C6-7
MacGee [11]	1979	16	F	C2 posterior arch
Palmer and Blum [12]	1980	14	M	C7 facet joint
Khosla et al. [13]	1999	17	M	C7 lamina
Sharma et al. [14]	2002	18	M	C1 posterior arch
Kulkarni et al. [15]	2004	15	M	T10-11 facet joint
McCall et al. [16]	2006	13	F	C3 lamina
Moon et al. [17]	2006	16	M	C5-6 lamina
Samartzis and Marco [18]	2006	11	M	S2 lamina
Sil et al. [9]	2006	6	F	C6-T2 pedicles
Song and Lee [19]	2007	11	M	T4 articular process
Srikantha et al. [20]	2008	17	M	C3 spinolaminar junction
Wang & Chou [21]	2009	16	F	C1 anterior arch
Lotfinia et al. [22]	2010	17	M	L3 inferior facet joint
Mudumba and Mamindla [23]	2012	14	M	C3 lamina
Rahman et al. [24]	2012	16	M	C1 posterior arch
Zaijun et al. [5]	2013	11	M	T1-7 lamina
		15	M	C2-3 lamina
		16	M	T5-6 spinous + transverse process
		17	F	T6 pedicle
Zinna and Chow [25]	2013	9	M	C2 arch
Mardi and Madan [26]	2013	9	M	T1 body + posterior arch
Pourtaheri et al. [27]	2014	11	M	L3 inferior articular process
Sultan et al. [8]	2016	8	M	C1 posterior arch
Raswan et al. [7]	2017	16	M	C3 posterior arch
Ganesh et al. [28]	2018	9	M	T4 facet joint + pedicle
Pawar et al. [4]	2020	6	F	T12 lamina + spinous process

The pathogenesis of osteochondroma remains unknown. Theories revolve around the pathologic microfractures of the epiphyseal growth plate leading to cartilaginous proliferation and endochondral ossification [3]. The cervical spine is the most common location of spinal lesions [2,10]. Our review of the current literature revealed that approximately 56% of lesions involve the cervical spine, following general trends seen for all age groups. Increased mobility of the cervical spine and a history of trauma have been proposed as contributing factors in the pathogenesis of osteochondroma [3,7]. An inciting incident may be difficult to identify, with many cases presenting years after the event. However, our patient’s history of a fall on her right side two years before diagnosis raises suspicion of the fall as the inciting incident.

The duration and speed of secondary center ossification in the cervical spine compared to other segments is thought to contribute to the increased incidence in this spinal region [4]. The speed of ossification is the fastest in the superior segments of the spine and decreases inferiorly, resulting in the cervical spine being at the highest risk for aberrant growths [8]. The average age of pediatric osteochondroma with cord compression in our literature review is about 13 years. The average appearance of secondary ossification centers between ages 11 and 18, further supporting their role in disease pathogenesis.

CT is the most accurate imaging modality for the localization of spinal osteochondromas. MRI can be used in conjunction to evaluate the degree of spinal cord compression and involvement of the surrounding structures. To minimize radiation exposure in pediatric populations, imaging using MRI studies rather than CT scans should be considered if clinically appropriate. Surgical decompression via laminectomy and laminotomy is the most common treatment strategy. Fowler et al. provide a comprehensive review of surgical techniques and associated outcomes; however, the data are generalized to all age groups [3]. The need for spinal fusion should be assessed based on the level of instability following removal of the osteochondroma. Emphasis should be placed on complete resection of the cartilaginous cap to minimize the risk of recurrence. The diagnosis is confirmed pathologically with the microscopic presence of a cartilage cap continuous with the underlying bone. The patient should be closely followed to ensure the resolution of the neurological signs and symptoms. The majority of patients have complete resolution of their neurologic deficits.

## Conclusions

The evaluation of myelopathic symptoms in a nine-year-old girl revealed an osteochondroma of C4-6 with cord compression at the C4-5 level. In children with spinal cord compression due to what appears to be a bony lesion on imaging, osteochondroma should be considered in the differential diagnosis as these lesions are not as rare as once thought. Appropriate imaging followed by surgical decompression is key to minimizing the risk of permanent neurologic deficits, tumor recurrence, and, although rare, malignant transformation.
